# AOD–PM_2.5_ Association: Paciorek and Liu Respond

**DOI:** 10.1289/ehp.0901732R

**Published:** 2010-03

**Authors:** Christopher J. Paciorek, Yang Liu

**Affiliations:** Department of Biostatistics, Harvard School of Public Health, Boston, Massachusetts, E-mail: paciorek@hsph.harvard.edu; Department of Environmental and Occupational Health, Rollins School of Public Health, Emory University, Atlanta, Georgia

We thank Kumar for his letter about our article (Paciorek and Liu 2009). We hope this exchange will highlight some of the key issues in using aerosol optical depth (AOD) for air quality purposes, in particular with regard to our focus on epidemiologic use. More dialogue is needed between scientists involved in remote sensing and those studying air pollution exposure and its epidemiologic effects with regard to the challenges and needs involved in making the remote sensing products helpful in applications. Our response to Kumar’s letter highlights our perspective on these challenges.

We agree that in our article (Paciorek and Liu 2009) we used AOD in a relatively straightforward way, and we welcome more advanced approaches to making use of AOD; in fact, one of us (Y.L.) is heavily involved in such work. The scientific challenge is to ensure that more advanced techniques can be used over an entire continuous time period and spatial area needed in a given epidemiologic or regulatory context. In our analysis we did our best to make use of currently available AOD products and to adjust for meteorologic variables and large-scale spatial discrepancy between AOD and particulate matter ≤ 2.5 μm in aerodynamic diameter (PM_2.5_) based on the data available. More sophisticated approaches will hopefully reduce the discrepancy between PM_2.5_ and AOD, but this does not change the need for rigorous assessment of the use of AOD as a proxy for PM_2.5_. An important test—which we explored—is the ability of AOD to help improve PM_2.5_ predictions, beyond reporting correlations between AOD and PM_2.5_. Furthermore, even with improved approaches in which systematic discrepancy may be alleviated, systematic discrepancy seems unlikely to disappear, and we believe serious consideration of AOD as a proxy for PM_2.5_ in the future will need to consider the nature of this discrepancy and its implications for the contexts in which AOD is used as a proxy for PM_2.5_.

To the extent that natural sources of AOD do not correlate with concentrations of ground-level PM_2.5_, we agree with Kumar that it would be ideal to control for such sources. We used the standard MODIS (moderate resolution imaging spectroradiometer) AOD product because this product would be available to general users; however, it would be appealing if a more tailored AOD retrieval algorithm could be applied over the spatial and temporal domain of interest for a given application. From reading the article by Kumar et al. (2008; particularly p. 3390), we did not see a specific algorithm proposed to decompose AOD into anthropogenic and natural sources or to control for natural sources.

As noted by Kumar, averaging all data—rather than matching in time before averaging—reduces associations. However, when interest lies in developing a proxy for long-term average PM_2.5_, the average of all monitoring data available at a regular interval should be an unbiased estimate of true PM_2.5_ at the location, which is the quantity one would like to have everywhere in space. Estimated associations based on matched data therefore are an overly optimistic assessment of AOD as a proxy for true long-term PM_2.5_. Of course for shorter intervals, the variability in estimates of true PM_2.5_ that are based on small numbers of daily samples will contribute to reduced AOD—PM_2.5_ association, so there are tradeoffs in deciding whether to match. One also needs to consider whether using matching introduces bias because missing AOD is associated with particular meteorologic conditions that also likely correlate with PM_2.5_ levels (Liu et al. 2009; Paciorek et al. 2008). Finally, in unpublished work, we have seen moderate improvements in associations when matching, but these improvements were not so large as to suggest that lack of time-matching is the key reason for the results seen in our article (Paciorek and Liu 2009). The reference to results on the diminishing association with longer-term aggregation by Kumar et al. (2007) seems to reinforce our very point: One should be cautious about using AOD as a proxy for PM_2.5_ when aggregating over time, but this is precisely one of the contexts in which we need proxies for PM_2.5_. Health analyses do not have the luxury of only analyzing health outcomes that correspond to the time periods (and spatial locations) for which AOD is available or for which AOD is thought to be a more reliable proxy.

Given the pixel-scale AOD retrievals (and the changing MODIS pixels from day to day), to spatially align our various data sources we took the ad hoc approach of assigning to each 4-km grid cell the value of the nearest MODIS AOD pixel overlapping the grid cell, requiring the distance between the cell and pixel centroids to be no greater than the nominal distance between AOD pixel centroids. This does not fundamentally change the AOD spatial pattern but does somewhat blur the original AOD values at the pixel boundaries. We recognize that it is difficult to compare a pixel-wide AOD value to a point observation of PM_2.5_, and of course one cannot expect AOD to provide information below its nominal resolution. Given this, in our statistical modeling we did our best to account for local sources of variation in PM_2.5_, namely distance to roads and to point sources, that cause the point-level observations to necessarily differ from the pixel-scale AOD. One would hope that the AOD pixel value represents variation in AOD at the scale of pixels or at somewhat larger resolution (such as distinguishing variation at scales up to 50–100 km) that differentiates urban, suburban, and rural areas. We would like variation at this scale to provide information on PM_2.5_ variation at the same scale that would improve prediction of PM_2.5_, but our results unfortunately did not provide evidence for such improvement. It is not completely clear what Kumar is suggesting as an alternative to using the value of AOD assigned to a pixel as representative of the entire pixel area, but it seems to be an approach that uses subpixel-scale information not available in the current MODIS AOD product. This seems promising, and we welcome work on providing AOD at higher resolution and evaluating whether more highly resolved AOD improves predictions of PM_2.5_. A key issue from this perspective is not the nominal resolution at which AOD is provided but the resolutions at which it is associated with spatial variations in PM_2.5_.

It was not entirely clear what Kumar is suggesting in terms of how to control for natural-source AOD and its spatio-temporal structure. With regard to large-scale discrepancy between AOD and PM_2.5_ that might mask smaller-scale correspondence, we used AOD and PM_2.5_ data to estimate and adjust (our calibrated AOD) for large-scale spatial discrepancy that persists over time but found that this did not improve matters, suggesting that small-scale discrepancy between PM_2.5_ and AOD is a major concern.
